# Advancing antimicrobial therapy: evaluating the ASTar (Q-linea) System for rapid AST in Gram-negative bloodstream infections

**DOI:** 10.1128/spectrum.03581-25

**Published:** 2026-04-20

**Authors:** Shikha Chaudhary, Noah Henry, Paula D. Binsol, Monish Sheth, Sangeetha Ranganath, John K. Midturi, Tove Goldson, Tiffany LaDow, Arundhati Rao, Manohar Mutnal

**Affiliations:** 1 Department of Pathology, Baylor Scott and White Medical Center 7866, Temple, Texas, USA; 2 Department of Medicine, Baylor Scott and White Medical Center 7866, Temple, Texas, USA; 3 Department of Medicine, Division of Infectious Diseases, Baylor Scott and White Medical Center 7866, Temple, Texas, USA; 4 Department of Medicine, Baylor Scott and White Medical Center, Waco, Texas, USA; 5 Infectious Diseases Pharmacist, Baylor Scott and White Medical Center 7866, Temple, Texas, USA; The University of Texas at Tyler, Tyler, Texas, USA

**Keywords:** bloodstream infections, rapid antimicrobial susceptibility testing, ASTar System (Q-linea), microscan walkaway, clinical impact, length of stay

## Abstract

Bloodstream infections (BSIs) caused by Gram-negative bacteria are a leading cause of sepsis and mortality, underscoring the need for rapid, reliable antimicrobial susceptibility testing (AST) to guide targeted therapy. We evaluated the analytical performance, turnaround time, and potential clinical impact of the ASTar System (Q-linea), an automated rapid phenotypic AST platform that reports minimum inhibitory concentration-based results directly from positive blood cultures. A total of 76 prospective clinical and 32 antibiotic-resistant specimens from the CDC & FDA AR Isolate Bank selected to represent diverse and well-characterized resistance profiles were tested and compared with the Standard-of-Care (SoC) MicroScan-WalkAway method using 2021 FDA STIC breakpoints. ASTar demonstrated essential and categorical agreement of 98.4% and 99.3% with SoC for clinical isolates, and 95.4% and 97.0% for CDC & FDA AR Isolate Bank specimens, respectively. The median turnaround time for ASTar results was 13.1 h compared with 51.2 h for SoC (*P* < 0.0001). Clinician-led retrospective chart review indicated earlier ASTar results could have influenced antimicrobial therapy in 83% of cases, enabling de-escalation in 66.2%, escalation in 13.8%, adjustment of route of administration in 15.4%, potential dose adjustment in 1.5%, and confirmation of the ongoing empiric therapy in 16.9%, with anticipated benefits, such as reduced antibiotic-related side effects in 47.7%, decreased antibiotic exposure in 46.2%, earlier discharge on oral therapy in 32.3%, correction of ineffective empiric therapy, and reduced length of stay, were each anticipated in 21.5% and 15.4% of cases, respectively. These findings support the use of ASTar to improve the management of Gram-negative BSIs through faster, targeted antimicrobial therapy.

**IMPORTANCE:**

Delays in antimicrobial susceptibility testing for Gram-negative bloodstream infections prolong empiric broad-spectrum therapy and can worsen clinical outcomes. This study presents data on the analytical performance, turnaround time, and potential clinical impact of the ASTar System for rapid antimicrobial susceptibility testing of Gram-negative bloodstream infections. The comparison of ASTar results with a Standard-of-Care method offers data to support clinical laboratories in assessing rapid phenotypic AST platforms, such as ASTar, their role in antimicrobial stewardship, and possible workflow adjustments for faster, targeted therapy in the management of Gram-negative bloodstream infections.

## INTRODUCTION

Bloodstream infections (BSIs) caused by Gram-negative bacteria are a leading cause of sepsis, multi-organ dysfunction, and death, with mortality rates ranging from 20% to 50%, depending on patient population and infection severity (1–6). In clinical practice, diagnosis and treatment rely on blood cultures followed by organism identification and AST. However, conventional methods, such as culture-based phenotypic antimicrobial susceptibility testing (AST) or broth microdilution, although considered the gold standard, typically require 24–48 h after blood culture positivity to generate susceptibility results (7, 8). This delay often necessitates empiric broad-spectrum antimicrobial use, which can lead to prolonged inadequate therapy, increased risk of adverse events, and longer hospital stays (6, 9, 10).

Recent advances in rapid AST platforms aim to address this critical gap by providing phenotypic susceptibility results directly from positive blood cultures within hours. Although molecular approaches can quickly detect selected resistance genes, they cannot determine minimum inhibitory concentrations (MICs) or full phenotypic susceptibility patterns (11). In contrast, rapid phenotypic systems overcome these limitations by directly measuring bacterial growth in the presence of antimicrobial agents, producing MIC-based results that are clinically actionable (12).

The ASTar System (Q-linea, Uppsala, Sweden) is an automated rapid phenotypic AST platform that reports MIC-based susceptibility results in approximately 6 h by monitoring bacterial growth kinetics through time-lapse microscopy (13). In this study, we evaluated the ASTar system’s analytical performance, turnaround time (TAT), and potential clinical impact in a clinical microbiology laboratory. To ensure a comprehensive assessment across the full spectrum of susceptibility and resistance, the evaluation incorporated Gram-negative bacteria from two sources: prospectively collected patient-derived positive blood cultures and contrived specimens that were well-characterized antibiotic-resistant (AR) isolates obtained from the CDC & FDA AR Isolate Bank. These AR isolates, selected for their diverse resistance profiles, were intentionally included to rigorously challenge the ASTar System using the investigational use-only (IUO) panel.

## MATERIALS AND METHODS

### Study design and objectives

This study aimed to evaluate the diagnostic performance of the ASTar System (Q-linea, Uppsala, Sweden) for antimicrobial susceptibility testing of Gram-negative bacterial pathogens in BSIs. The primary objectives were to assess the analytical performance, TAT, and potential clinical impact of the ASTar System by comparing its performance with the SoC method, the MicroScan WalkAway System (Beckman Coulter, CA, USA).

The study included 76 prospectively collected patient-derived positive blood cultures from individuals with Gram-negative BSIs and 32 AR specimens from the CDC & FDA Antibiotic Resistance Isolate Bank (https://wwwn.cdc.gov/arisolatebank/), totaling 108 Gram-negative specimens. The AR isolates were specifically selected to represent clinically relevant resistance mechanisms, such as extended-spectrum β-lactamase (ESBL) production and carbapenem-mediated resistance (Table S1). This inclusion of the CDC & FDA AR Isolate Bank specimens ensured evaluation of the ASTar System’s performance across a varied spectrum of resistance phenotypic profiles.

All positive blood culture specimens were tested on the ASTar and SoC instrument against a panel of 23 clinically relevant antimicrobial agents (Table 1). The analytical performance of the ASTar system was assessed by comparing minimum inhibitory concentrations (MICs), essential agreement (EA), categorical agreement (CA), very major errors (VME), and major errors (ME) as per the 2021 FDA STIC breakpoints (14, 15). The study also compared the TAT of the ASTar and the SoC method. The TAT was defined as the interval between the positive blood culture signal and the availability of MIC results that would have led to a change in therapy. Turnaround time (TAT) analysis was performed on 69 patient-derived specimens for which ASTar and standard-of-care (SoC) testing were conducted from the same blood culture bottle. The potential clinical impact analysis was limited to cases with complete medical records available for review (*n* = 65).

**TABLE 1:** A panel of 23 antimicrobial agents tested for susceptibility in this study.

S. no.	Antibiotic
1	Amikacin
2	Ampicillin
3	Ampicillin-sulbactam
4	Aztreonam
5	Cefazolin
6	Cefepime
7	Cefotaxime
8	Cefoxitin
9	Ceftazidime
10	Ceftazidime-avibactam
11	Ceftolozane-tazobactam
12	Ceftriaxone
13	Cefuroxime
14	Ciprofloxacin
15	Ertapenem
16	Gentamicin
17	Levofloxacin
18	Meropenem
19	Meropenem-vaborbactam
20	Piperacillin-tazobactam
21	Tigecycline
22	Tobramycin
23	Trimethoprim-sulfamethoxazole

For each patient, TAT from both ASTar and SoC results was compared, with two-sample *t*-tests used to compare the means. Additionally, a clinician-led retrospective chart review was conducted to assess the potential clinical impact of earlier ASTar results on therapy decisions and patient outcomes.

### Patient enrollment: inclusion and exclusion criteria

Eligible participants were adult patients aged 18 to 80 years diagnosed with Gram-negative monomicrobial BSIs at BSWH. Enrollment occurred within 16 h of blood culture positivity, as flagged by the automated BacT/ALERT system (bioMérieux, Marcy-l’Étoile, France). Only infections caused by the organisms on the ASTar IUO panel (listed in Table S2) were included in the study. Patients were excluded if blood culture data could not be retrieved due to transfer to another ward or location, if polymicrobial infection was identified by Gram staining, if death of the patient occurred within 24 h of blood culture positivity, if the infection was caused by an off-panel organism not represented in the ASTar IUO panel, or if one or more of the inclusion criteria were not met.

### Sample collection and pathogen identification

A total of 76 Gram-negative bacterial positive blood cultures were prospectively collected from adult patients with BSIs and included in this study. Specimens were identified upon detection of a positive signal by the automated blood culture monitoring system (BacT/ALERT), followed by Gram staining to confirm the presence of Gram-negative rods. Species-level identification was then performed using the Verigene System (Nanosphere, Northbrook, IL, USA) and the resulting identifications were used for the study.

In addition, 32 well-characterized antimicrobial resistant specimens from the CDC & FDA AR Isolate Bank were contrived into sterile blood culture bottles to simulate bloodstream infections. These isolates were selected to represent diverse resistance phenotypes and were prepared and inoculated following a standardized spiking protocol (Table S1).

Briefly, fresh cultures of each AR isolate were grown on Blood Agar Plates (BAPs) and examined to confirm sufficient growth, correct colony morphology, and purity. Colonies were suspended in 3 mL of sterile saline to prepare an initial 0.5 McFarland standard suspension (~1.5 × 10⁸ CFU/mL), which was thoroughly mixed by vortexing. This suspension was then serially diluted in two steps: first, 0.1 mL of the McFarland suspension was added to 10 mL of sterile saline to create a 1:100 dilution (~1.5 × 10⁶ CFU/mL); then, 0.1 mL of this dilution was further added to another 10 mL of sterile saline to achieve the final working suspension (~1.5 × 10⁴ CFU/mL). From the final dilution, 0.1 mL (~1,500  CFU) was inoculated under aseptic conditions into a BacT/ALERT blood culture bottle containing 8–10 mL of freshly collected human whole blood, after disinfecting the bottle top with 70% isopropyl alcohol. The inoculated bottles were then incubated in the BacT/ALERT system until flagged positive. Gram staining and species-level identification of contrived specimens were performed in the same manner as clinical specimens to ensure appropriate recovery and identity. Each confirmed culture, whether clinical or contrived, was tested in parallel using both the ASTar System and the SoC MicroScan WalkAway method.

### ASTar System workflow

The ASTar System is an automated platform designed to provide rapid MIC-based susceptibility results directly from positive blood culture bottles. For each enrolled specimen, approximately 1 mL of positive blood culture was transferred into the ASTar BC G-Kit (IUO panel) cartridge. A frozen insert (stored at −20°C) was inserted into the cartridge, and the specimen was then loaded on the instrument along with the ASTar BC G-disc. The process required approximately 1–2 min of hands-on time per sample. The instrument performed automated incubation and real-time monitoring of bacterial growth in the presence of antimicrobial agents. Identification of microorganism data could be manually entered at any point during the process. The system determined MIC values and interpreted susceptibility according to FDA STIC breakpoints (15). Reported TAT reflects routine laboratory workflow and timing of specimen enrollment.

### Comparator method and discrepancy resolution

The MicroScan WalkAway system was used as the reference SoC method. Essential and categorical interpretations (susceptible, intermediate, resistant) from the ASTar System were compared with the SoC results for select antimicrobials available on both the panels to assess concordance. The evaluation included measures of EA for MIC values, CA for interpretive categories, identification of discrepancies and errors, such as VME and ME. Discrepancies between the two methods were further evaluated using broth microdilution (BMD) testing, performed in triplicate, using Sensititre Panels (GN7F, STP8F) (Thermo Fisher Scientific, Waltham, MA, USA). The modal MIC value from BMD was used as the adjudicated result. The specific isolates that underwent discrepancy resolution testing are listed in Table S3.

### Quality control

A series of quality control (QC) strains were utilized to ensure the proper functioning of the ASTar System as part of the routine QC schedule. The QC strains included *Escherichia coli* ATCC 25922, *Pseudomonas aeruginosa* ATCC 27853, *Klebsiella pneumoniae* ATCC 700603, and *Klebsiella pneumoniae* ATCC BAA 2814. These strains were processed according to the standard operating procedures for quality control and were integral in validating the accuracy and consistency of the ASTar System’s performance.

### Potential clinical impact assessment

To evaluate the potential clinical utility of integrating the ASTar System into routine patient management, a hypothetical clinical impact assessment was conducted by the care team. Only clinical patient-derived positive blood culture specimens with complete and traceable medical records were included in this analysis (*n* = 65). No additional patient selection criteria were applied. This assessment included only the clinical patient-derived positive blood culture specimens while contrived CDC & FDA AR Isolate Bank specimens were excluded from this analysis.

The chart reviews were conducted retrospectively by a multidisciplinary team comprising three infectious disease (ID) physicians, one ID clinical pharmacist, and one hospitalist. For each of the 65 evaluable cases with complete records, the team was presented with an ASTar susceptibility report generated directly from the positive blood culture, along with a structured electronic survey summarizing patient demographics, clinical condition, and the empiric antimicrobial regimen at the time of blood culture positivity.

Clinicians were asked to review each case independently, without access to the SoC susceptibility data, and to determine whether earlier availability of ASTar results would have influenced their antimicrobial management. In addition, they were asked to identify any potential downstream clinical outcomes that could have been affected by such modifications. Only cases with complete medical records at the time of review were included in the analysis. All responses were hypothetical and non-interventional, intended to simulate potential clinical decision-making informed by rapid AST data.

## RESULTS

### Sample collection and organism distribution

**Fig 1:**
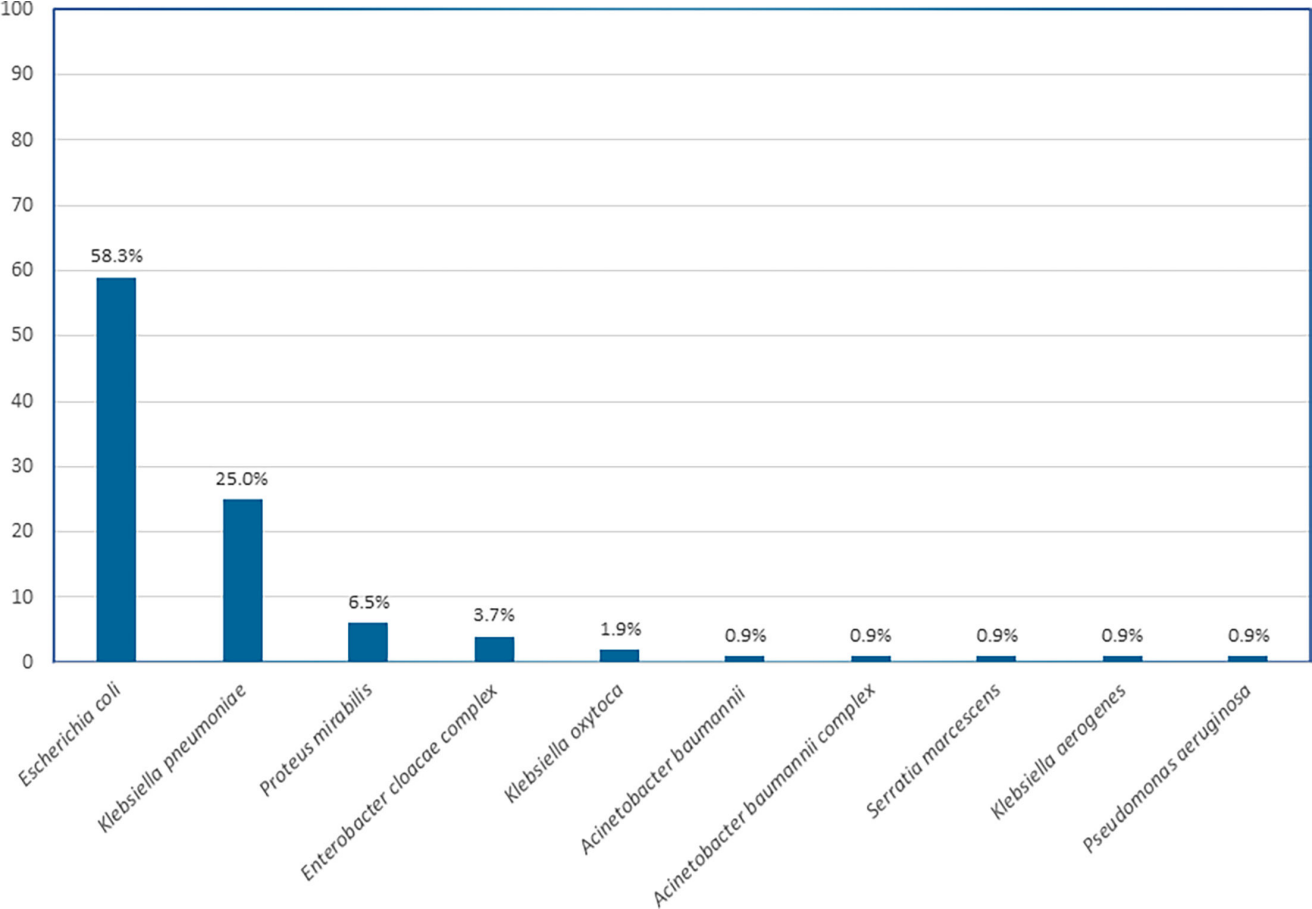
Distribution of identified Gram-negative organisms from *n* = 108 clinical patient-derived and CDC & FDA AR Isolate Bank positive blood culture specimens included in the study. Bars represent the proportion of each organism as a percentage of the total specimens. The most common species identified were *Escherichia coli* (58.3%) and *Klebsiella pneumoniae* (25.0%), followed by *Proteus mirabilis* (6.5%), *Enterobacter cloacae complex* (3.7%), *Klebsiella oxytoca* (1.9%), *Acinetobacter baumannii* (0.9%), *Acinetobacter baumannii complex* (0.9%), *Serratia marcescens* (0.9%), *Klebsiella aerogenes* (0.9%), and *Pseudomonas aeruginosa* (0.9%).

We tested 76 clinical, patient-derived Gram-negative bacterial positive blood culture specimens and 32 well-characterized antibiotic-resistant (AR) bank specimens, selected to ensure a broad spectrum of resistance phenotypes, for a combined total of 108 Gram-negative BSI specimens. The organisms identified among these blood culture specimens were 63/108 *Escherichia coli* (58.3%), 27/108 *Klebsiella pneumoniae* (25.0%), 7/108 *Proteus mirabilis* (6.5%), 4/108 *Enterobacter cloacae complex* (3.7%), 2/108 *Klebsiella oxytoca* (1.9%), 1/108 *Acinetobacter baumannii* (0.9%), 1/108 *Acinetobacter baumannii complex* (0.9%), 1/108 *Serratia marcescens* (0.9%), 1/108 *Klebsiella aerogenes* (0.9%), and 1/108 *Pseudomonas aeruginosa* (0.9%) (Fig. 1).

**Fig 2:**
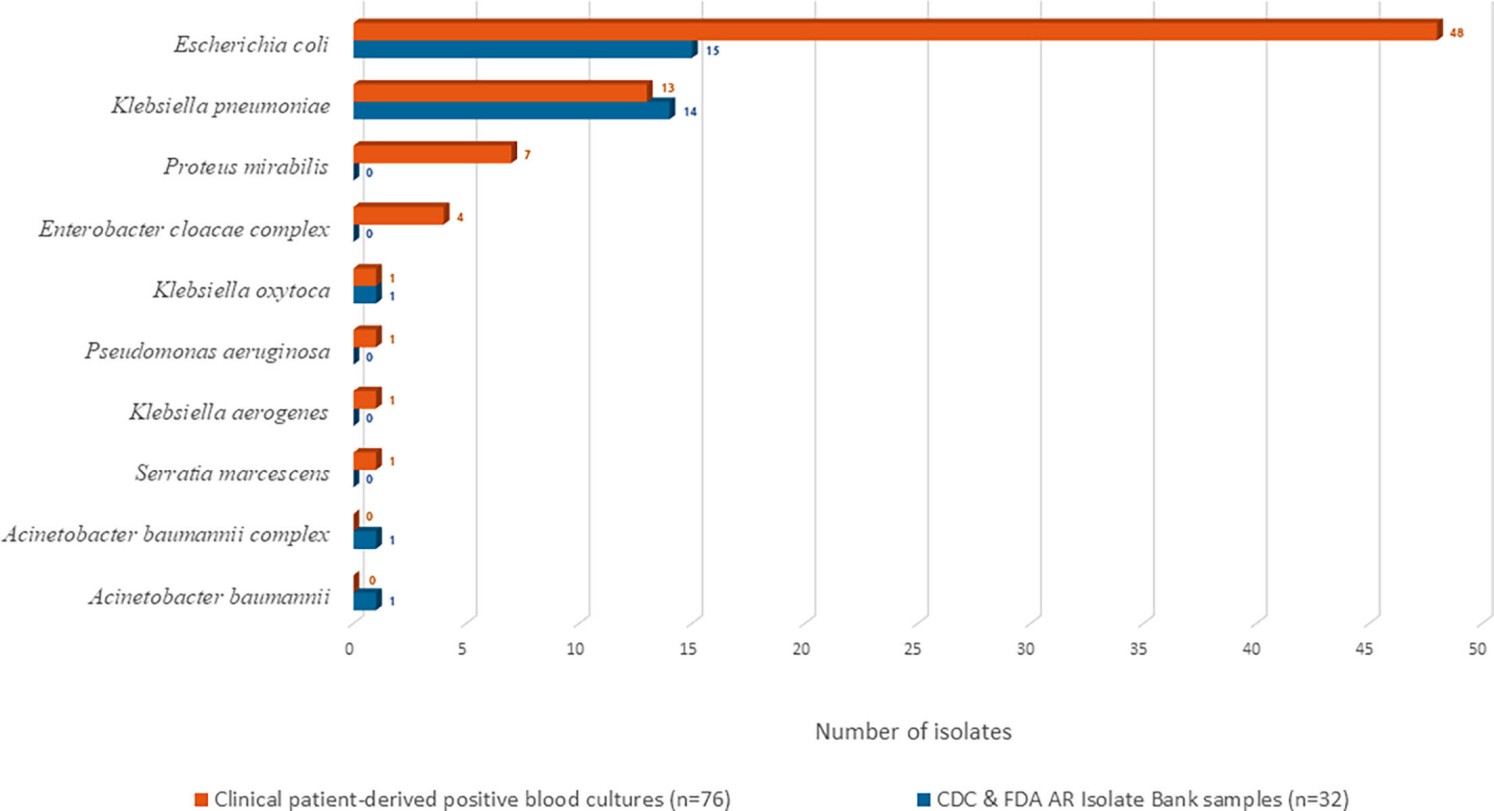
Distribution of Gram-negative organisms identified from clinical patient-derived and CDC & FDA AR Isolate Bank-contrived positive blood culture specimens. The horizontal bar chart depicts the number of specimens by blood source: clinical patient-derived positive blood cultures (*n* = 76; orange bars) and contrived CDC & FDA AR Isolate Bank specimens (*n* = 32; blue bars). *Escherichia coli* and *Klebsiella pneumoniae* were the most common species in both groups, with additional less frequent species shown.

The detailed distribution of organisms across the two specimen sets, 76 clinical patient-derived isolates and 32 CDC & FDA AR Isolate Bank isolates, is described in Fig. 2. The AST was performed on all isolates using the ASTar System against a panel of 23 antibiotics, resulting in 1,691 organism-antibiotic combinations for 76 clinical patient-derived positive blood cultures and 687 combinations for 32 CDC & FDA AR Isolate Bank specimens. The ASTar System results were compared with those obtained using the SoC method, the MicroScan WalkAway system.

### AST performance

We evaluated the analytical performance of the ASTar System against the SoC using EA, CA, and rates of VME and ME. These metrics provide a standardized framework for evaluating the accuracy and reliability of a new susceptibility testing platform (14). For 76 clinical positive blood culture specimens, the ASTar System demonstrated high concordance with the SoC method, achieving an EA of 98.4% and a CA of 99.3%, with 0.1% ME and no VME. For 32 contrived CDC & FDA AR Isolate Bank specimens, the ASTar System showed an EA of 95.4% and a CA of 97.0%, with VME and ME rates of 1.4% and 3.7%, respectively (Table 2). EA, CA, VME, and ME rates per antibiotic after discrepancy resolution are provided in Tables S4 and S5 .

**TABLE 2:** Analytical performance of the ASTar System compared with the Standard-of-Care (SoC) MicroScan WalkAway method^*a*^

	EA (%)	CA (%)	VME (%)	ME (%)
Clinical patient-derived positive blood culture specimens (*n* = 76)	98.4	99.3	0.0	0.1
CDC & FDA AR Isolate Bank specimens (*n* = 32)	95.4	97.0	1.4	3.7

^*a*^ Essential agreement (EA), categorical agreement (CA), very major error (VME), and Major Error (ME) rates are shown separately for clinical patient-derived positive blood culture specimens (*n* = 76) and contrived CDC & FDA AR Isolate Bank specimens (*n* = 32).

### Turnaround time

**Fig 3:**
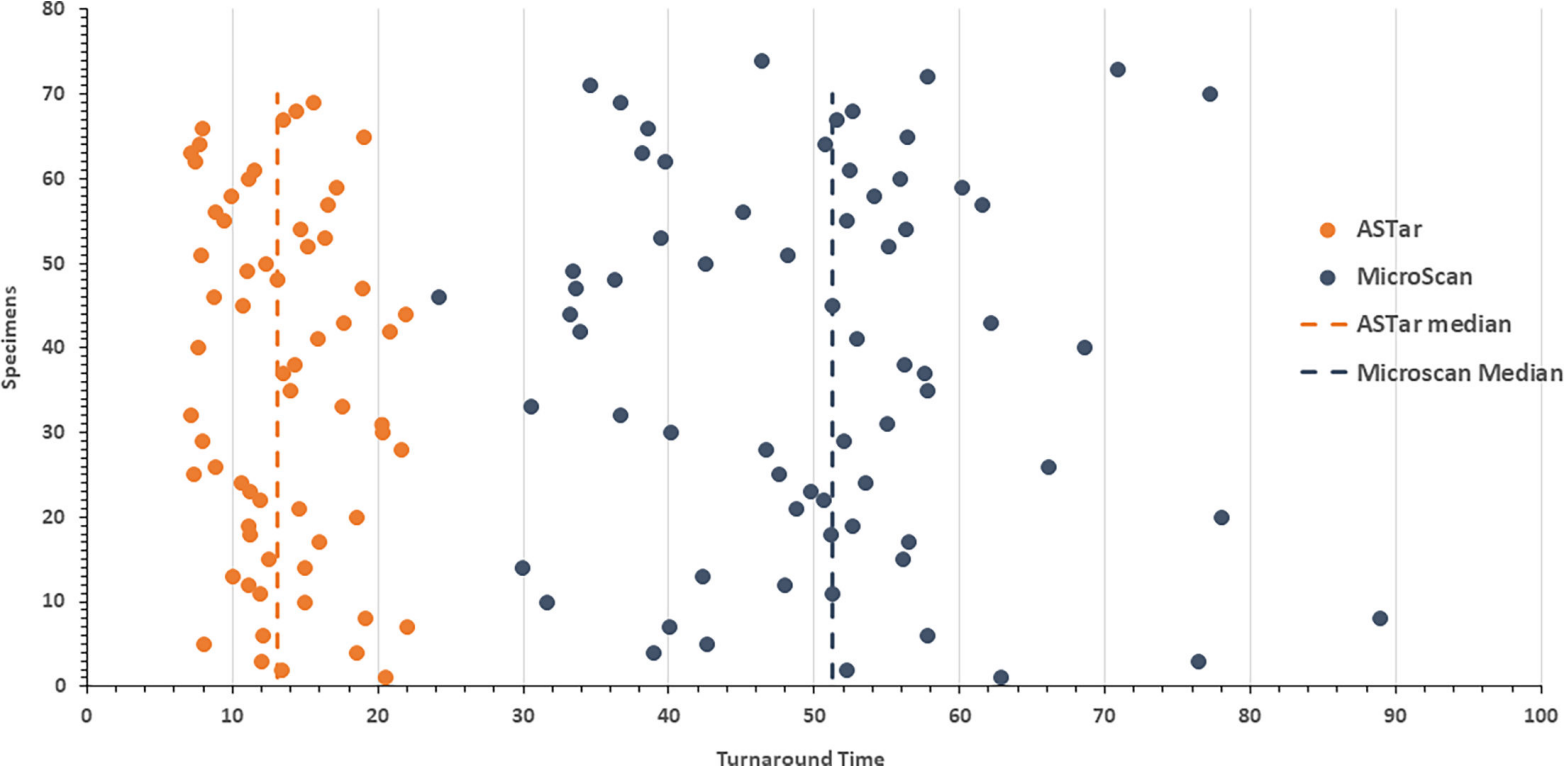
Turnaround time comparison for antimicrobial susceptibility testing using the ASTar system and the SoC MicroScan WalkAway system for *n* = 69 clinical Gram-negative blood culture specimens. Each point represents the TAT for a single specimen tested with ASTar (orange) and MicroScan (blue). Vertical dashed lines indicate the median TAT for each method: ASTar, 13.1 h (orange dashed line); MicroScan, 51.2 h (blue dashed line).

We compared the TAT of the ASTar System with that of the SoC method to directly assess the difference in TAT. A total of 69 clinical patient-derived positive blood culture specimens had complete time records and were included in the TAT analysis. The ASTar System demonstrated a faster TAT when compared to the SoC method with an average (SD) of 13.5 (4.2) h compared with SoC method average (SD) 50.2 (12.4) h, *P* < 0.0001 (Fig. 3). The median TAT was 13.1 h with ASTar versus 51.2 h with SoC, representing a reduction of nearly 38 h.

### Potential clinical impact

To explore the potential of providing the actionable susceptibility data sooner, we conducted a hypothetical clinical impact assessment in 65 clinical patient-derived positive blood culture cases with complete medical records. The goal was to evaluate how the earlier availability of ASTar results could have influenced antimicrobial therapy decisions and patient outcomes. All assessments were hypothetical, non-interventional, and based solely on clinical judgment, as ASTar results were not available to guide actual patient treatment. This retrospective simulation was designed to model potential decision-making scenarios informed by ASTar data, illustrating the system’s capacity to optimize antimicrobial use and improve patient care.

#### Potential clinical impact of ASTar on therapeutic decision-making

**Fig 4:**
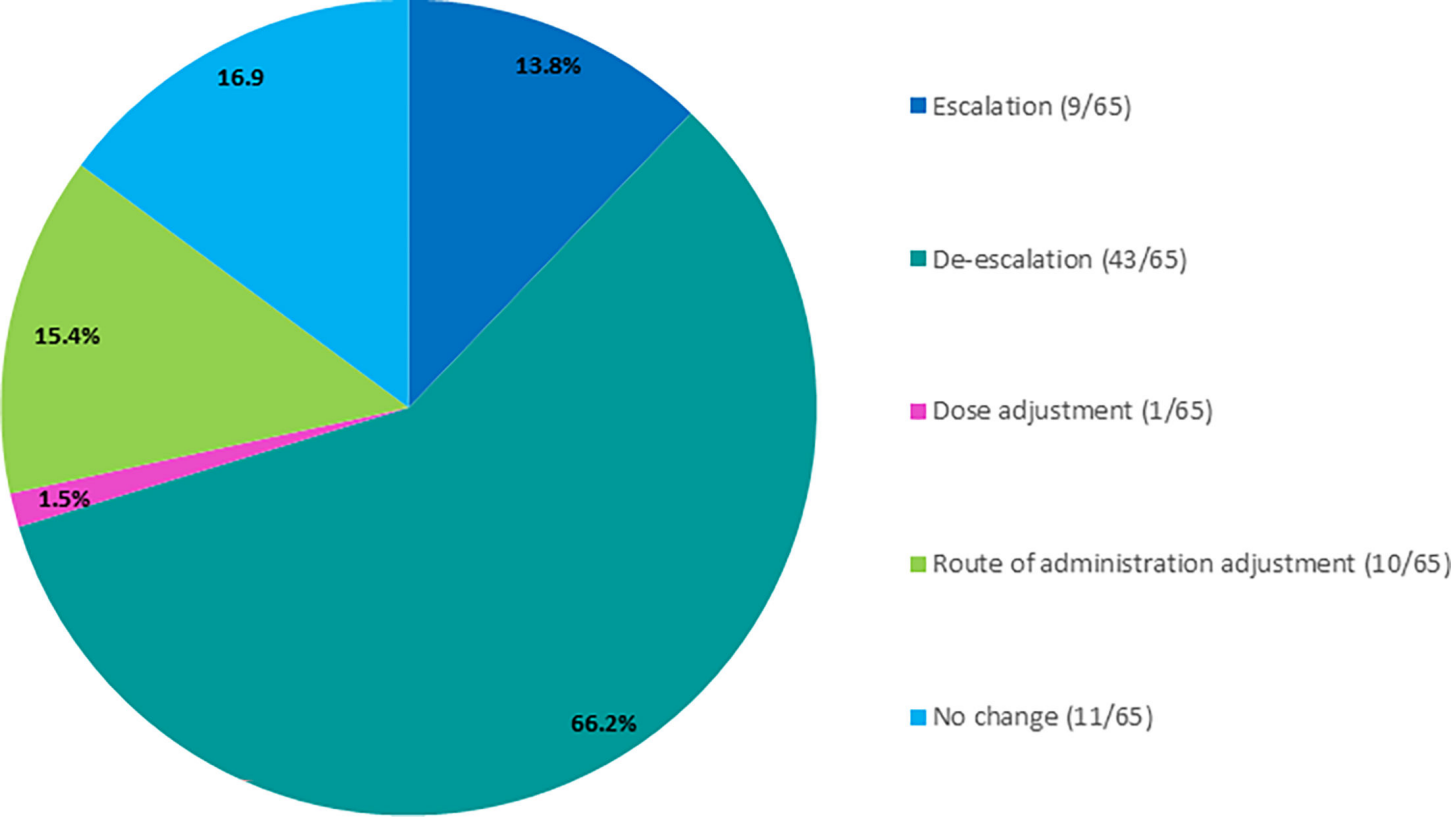
Distribution of potential clinical impact on change to empiric therapy based on ASTar-guided AST results (*n* = 65). ASTar could have enabled de-escalation of empiric therapy in 66.2% of cases, escalation in 13.8%, adjustment of route of administration in 15.4%, dose adjustment in 1.5% and confirmation of empirical treatment in 16.9% of cases. All responses were hypothetical and based on clinical judgment by ID physicians, an ID pharmacist, and a hospitalist. The total percentage is >100% since more than one clinical impact can apply.

As part of the hypothetical clinical impact assessment, a multidisciplinary team involving ID clinicians retrospectively reviewed each patient case and assessed how earlier availability of ASTar results might have altered their therapeutic decisions. This included evaluating whether they would have initiated a different antimicrobial agent, adjusted the spectrum of therapy, or modified the timing of escalation or de-escalation based on the earlier susceptibility data. Based on their retrospective review and clinical judgment, they indicated that having rapid ASTar results could have altered therapy in 54 of 65 (83%) of cases. The most frequently suggested changes included de-escalation of empiric broad-spectrum therapy in 43 of 65 cases (66.2%), switching the route of administration in 10 of 65 cases (15.4%), and escalation of therapy in 9 of 65 cases (13.8%). A potential dose adjustment was proposed in 1 of 65 cases (1.5%). In 11 of 65 cases (16.9%), clinicians anticipated no change to therapy even with faster results (Fig. 4). These findings underscore that, in most cases, clinicians believed that quicker AST results could have supported more targeted and appropriate antimicrobial management decisions.

#### Potential outcomes associated with ASTar-guided therapy modifications

**Fig 5:**
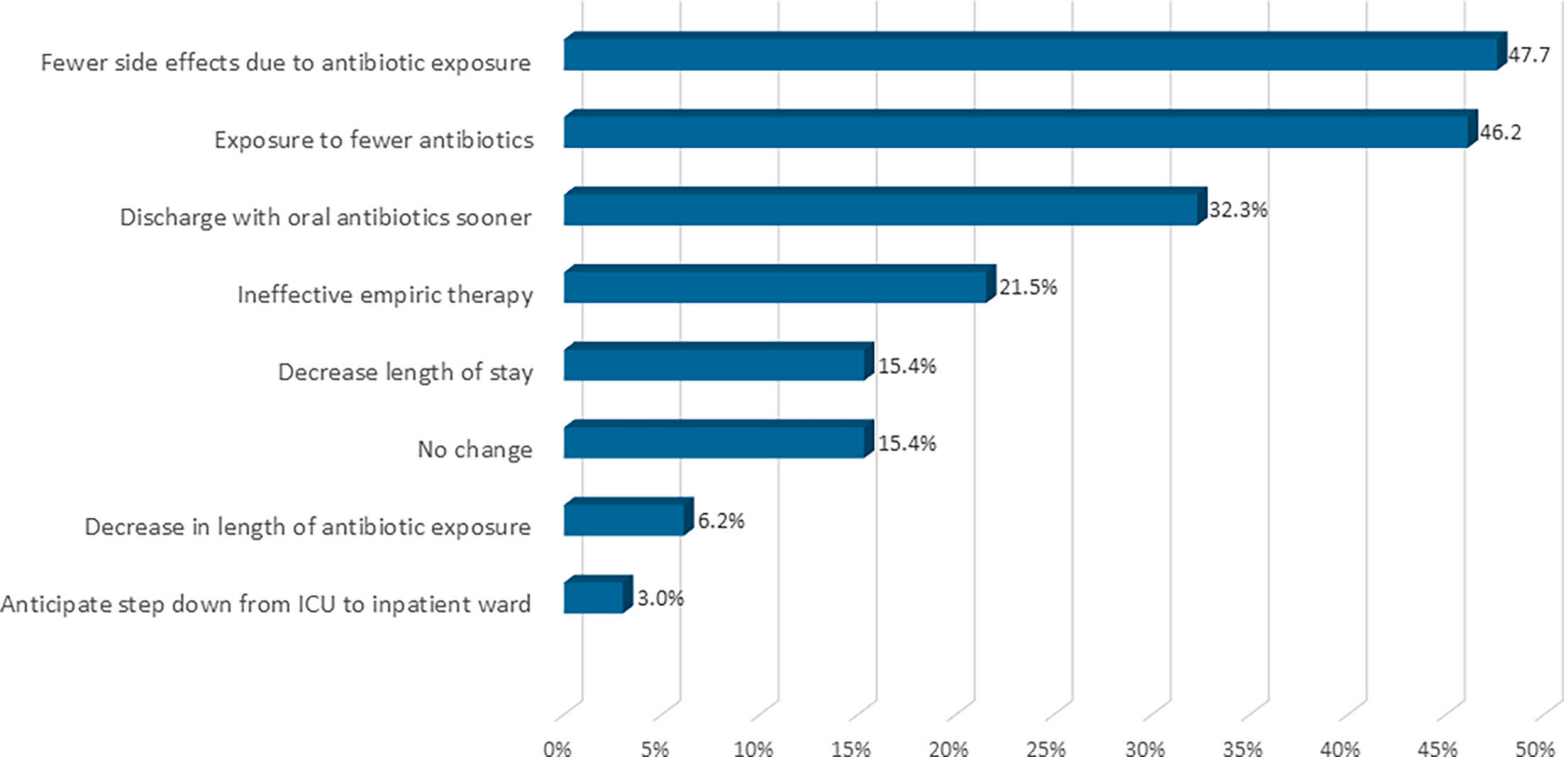
Potential clinical outcomes associated with ASTar-guided therapeutic modifications (*n* = 65). ASTar could have reduced antibiotic-related side effects in 47.7% of cases, decreased overall antibiotic exposure in 46.2%, and enabled earlier discharge on oral antibiotics in 32.3%. Correction of ineffective empiric therapy and reduced length of stay were each anticipated in 21.5% and 15.4% of cases, respectively. Smaller proportions were anticipated to benefit from shorter antibiotic duration in 6.2% cases, potential ICU step-down in 3.0% while no change was anticipated in 15.4%. All assessments were hypothetical and based on clinical judgment by ID physicians, an ID pharmacist, and a hospitalist. The total percentage is >100% since more than one clinical outcome can apply.

Clinician review of the most frequent hypothetical downstream clinical outcomes if therapy adjustments had been made based on earlier ASTar results included an anticipated reduction in antibiotic-related adverse effects in 47.7% cases and a decrease in overall antibiotic exposure in 46.2% cases. Earlier discharge on oral therapy was anticipated as a potential outcome in 32.3% of cases, correction of ineffective empiric therapy anticipated in 21.5%, while shortened hospital length of stay could have been possible in 15.4% cases. A reduction in total duration of antibiotic exposure was hypothetically anticipated in 6.2% of cases, and step-down from intensive care to an inpatient ward was considered feasible in 3.0%. Notably, in 15.4% of cases, clinicians anticipated no change in clinical outcomes despite earlier availability of ASTar results (Fig. 5).

## DISCUSSION

To the best of our knowledge, this is the first U.S. study to evaluate the analytical performance, TAT, and hypothetical potential clinical impact of the ASTar System compared with the MicroScan WalkAway as the SoC for AST testing of Gram-negative clinical patient-derived and CDC & FDA AR Isolate Bank specimens. The ASTar system demonstrated high concordance with MicroScan WalkAway, with EA and CA rates of 98.4% and 99.3%, respectively, with 0.1%ME and no VME for 76 clinical patient-derived positive blood cultures. These findings are consistent with a previous evaluation in Italy, which reported overall essential and categorical agreement rates of 98% and 96.1%, respectively, with major and very major error rates of 2.5% and 3.3%, respectively, when compared with the MicroScan reference method using EUCAST breakpoints (16).

In another similar Swedish study, the ASTar System demonstrated essential and categorical agreement rates of 95.8% and 97.6%, respectively, with major and very major error rates of 0.9% and 2.4%, respectively, when compared with BMD as the reference method using EUCAST breakpoints (17). Our findings are also consistent with a German study comparing ASTar results to standard BMD using EUCAST breakpoints, which reported essential and categorical agreement rates of approximately 90.7% and 95.6%, respectively, with major and very major error rates of 2.0% and 2.4%, respectively (12).

To rigorously evaluate the system’s ability to detect resistant phenotypes, the study also included a defined panel of 32 CDC & FDA AR Isolate Bank specimens. Resistant phenotypes represent some of the most challenging and clinically significant diagnostic scenarios, and so the use of these CDC & FDA AR Isolate Bank specimens allowed for a more comprehensive assessment of the ASTar’s ability to detect extended-spectrum beta-lactamases, carbapenemases, and other resistance mechanisms. The essential and categorical agreement rates for these specimens were somewhat lower than those observed for susceptible clinical specimens, at 95.4% and 97.0%, with very major and major error rates of 1.4% and 3.7%, respectively, yet still within the acceptable thresholds of EA and CA ≥90% (15). This slight reduction in performance for resistant strains reflects the greater diagnostic complexity of detecting extended-spectrum β-lactamases (ESBLs), carbapenemases, and other resistance mechanisms. Similar challenges have been reported with other AST platforms, such as BD Phoenix, Vitek 2, and MicroScan WalkAway Plus, particularly for piperacillin/tazobactam susceptibility testing in challenging *E. coli* and *Enterobacterales* isolates, where automated systems showed poor performance to CLSI breakpoints compared with BMD (18, 19).

This study used 2021 FDA STIC breakpoints; however, it is important to note that the FDA and CLSI breakpoints are not always aligned, which may influence susceptibility interpretation—particularly for resistant organisms. A recent publication reported over 100 differences between CLSI and FDA breakpoints as of 2024, including cases where FDA had not recognized any breakpoint for agents commonly used in clinical practice (20). Consequently, many clinical laboratories continue to follow CLSI guidelines, which can differ from FDA-cleared AST systems that have yet to incorporate updated breakpoints.

Often, in bloodstream infections, timely availability of AST results is critical for guiding appropriate antimicrobial therapy and improving patient outcomes. In addition to demonstrating high concordance and strong analytical performance, the ASTar System reduced the median TAT for susceptibility results. It provided susceptibility results in a median of 13.1 h from positive blood culture, compared with a median of 51.2 h for conventional methods, reducing the TAT by approximately 38 h compared with the SoC method. The average TAT with ASTar was 13.5 h as compared with 50.2 h for the SoC, potentially enabling susceptibility-guided therapy decisions more than a day earlier. These findings are consistent with previous evaluations of the ASTar and other rapid phenotypic AST platforms, such as Vitek Reveal, which have also demonstrated shortened TAT with varying degrees of agreement and workflow integration (12, 21). This acceleration is particularly critical in the management of BSIs, where delays in initiating effective antimicrobial therapy are strongly associated with increased mortality and morbidity (9, 22). Beyond the benefit of faster results, rapid AST also supports antimicrobial stewardship efforts by minimizing unnecessary broad-spectrum antibiotic use and facilitating earlier optimization of therapy (17, 23). Additionally, prior studies have shown that implementing rapid AST can reduce both the length of hospital stay and overall hospitalization costs, underscoring its clinical and economic value (24).

In many cases, delays in antimicrobial susceptibility testing compel clinicians to initiate broad-spectrum empiric therapy, which can result in suboptimal or inappropriate antimicrobial use. Rapid AST platforms have enabled more timely and targeted therapy and enhanced clinical outcomes in patients. The assessment of hypothetical clinical impact in this study indicated that earlier availability of ASTar results would have influenced therapeutic decisions in 83% of cases, most commonly through de-escalation of broad-spectrum therapy in 66.2% cases and importantly, escalation in 13.8% of cases. Reductions in antibiotic use, avoidance of unnecessary side effects, and earlier transition to oral therapy were among the anticipated benefits. These findings align with earlier studies showing improved antibiotic utilization when rapid AST is implemented alongside antimicrobial stewardship programs (13, 25).

Several limitations should be acknowledged. First, the single-center design may limit the generalizability of our findings. Second, the potential clinical impact evaluation was hypothetical with review of ASTar cases done by five clinicians, including ID physicians, an ID pharmacist, and a hospitalist. Future studies are needed to also explore cost-effectiveness and the development of automated reporting systems to enhance clinical actionability after actual implementation and use of rapid AST results.

In conclusion, the ASTar System demonstrated high analytical performance and a substantial reduction in TAT compared with the SoC method with the potential to improve timely optimal antibiotic therapy and associated outcomes. The inclusion of CDC & FDA AR Isolate Bank specimens confirmed its ability to detect resistant phenotypes with acceptable accuracy. These findings support the integration of rapid phenotypic AST systems into clinical microbiology and antimicrobial stewardship workflows to provide timely, reliable susceptibility results for Gram-negative BSIs.

### Conclusion

The ASTar System demonstrated comparable performance and efficiency for antimicrobial susceptibility testing of Gram-negative BSI positive blood culture specimens as compared with the SoC with the potential to improve timely optimal antibiotic therapy and associated outcomes. Essential and categorical agreement with the SoC MicroScan WalkAway system exceeded 95% for both clinical and CDC & FDA AR Isolate Bank specimen groups, with low rates of very major and major errors. The ASTar System reduced the TAT results by a median of approximately 38 h compared with the standard method. The reduced TAT was associated with a higher rate of hypothetical therapeutic adjustments, including escalation, de-escalation of broad-spectrum therapy, and changes in route of administration where indicated. These findings indicate that implementation of the ASTar System in clinical workflows holds the potential for earlier optimization of antimicrobial therapy for Gram-negative BSIs, while maintaining strong concordance with established SoC method.

## SUPPLEMENTAL MATERIAL

Tables S1 and S2 spectrum.03581-25-s0001.pdf Genotypic and phenotypic resistance profiles of CDC & FDA AR Isolate Bank isolates, and organisms included in the ASTar IUO panel.

Table S3 spectrum.03581-25-s0002.pdf Isolates with AST discrepancies that underwent resolution testing using Sensititre panels.

Tables S4 and S5 spectrum.03581-25-s0003.pdf Table S4: Essential agreement, categorical agreement, very major error, and major error rates by antimicrobial agent for ASTar vs Standard-of-Care method for 76 prospective clinical specimens, after discrepancy resolution. Table S5: Essential agreement, categorical agreement, very major error, and major error rates by antimicrobial agent for ASTar vs Standard-of-Care method for 32 CDC & FDA AR Isolate Bank samples, after discrepancy resolution.

ASM does not own the copyrights to Supplemental Material that may be linked to, or accessed through, an article. The authors have granted ASM a non-exclusive, world-wide license to publish the Supplemental Material files. Please contact the corresponding author directly for reuse.
