# Supplementary material for: Advancing antimicrobial therapy: evaluating the ASTar (Q-linea) System for rapid AST in Gram-negative bloodstream infections
Source: Microbiol Spectr. 2026 Apr 20;14(6):e03581-25. doi: 10.1128/spectrum.03581-25 (PMC13227951; doi:10.1128/spectrum.03581-25)
Supplement: Tables S1 and S2 — Genotypic and phenotypic resistance profiles of CDC & FDA AR Isolate Bank isolates, and organisms included in the ASTar IUO panel. [file spectrum.03581-25-s0001.pdf]

Supplementary Table 1. Genotypic and Phenotypic Resistance Profiles of CDC & FDA AR Isolate Bank Isolates (n=32)

S. no.	Strain	Genus	Species	Genotypic Resistance Profile	Phenotypic Resistance Profile
1.	AR0058	*Escherichia*	*coli*	TEM-20; aac(3)-IId, aadA5, aph(3")-Ib, aph(6)-Id; mph(A); sul1, sul2; tet(A); dfrA17; ACRF, qacEΔ1	Resistant to: ampicillin, ampicillin/sulbactam, aztreonam, cefazolin, cefepime, cefotaxime, cefoxitin, ceftazidime, ceftriaxone, gentamicin, levofloxacin, piperacillin/tazobactam, tetracycline, TMP/SMX
2.	AR0061	*Escherichia*	*coli*	KPC-3, OXA-9, TEM-1A; aac(6')-Ib, aadA1, aadA2, aph(3")-Ib, aph(6)-Id; sul1, sul2, sul3; tet(A), tet(R); dfrA12, dfrA14; EMRD, qacEΔ1	Resistant to: ampicillin, ampicillin/sulbactam, aztreonam, cefazolin, cefepime, cefotaxime, ceftazidime, ceftriaxone, ertapenem, imipenem, meropenem, gentamicin, piperacillin/tazobactam, tetracycline, TMP/SMX
3.	AR0069	*Escherichia*	*coli*	NDM-1, CMY-6, TEM-1; aac(6')-Ib3, aph(3")-Ib; sul1, sul2; tet(A), tet(R); dfrA8; ble-MBL, ACRF, EMRD, qacEΔ1	Resistant to: ampicillin, ampicillin/sulbactam, cefazolin, cefepime, cefotaxime, cefoxitin, ceftazidime, ceftriaxone, ertapenem, imipenem, meropenem, piperacillin/tazobactam, tetracycline, TMP/SMX

4.	AR0114	*Escherichia*	*coli*	KPC-3, TEM-1B; aadB, strA, strB; cmlA1; sul1, sul2; dfrA5	Resistant to: ampicillin, ampicillin/sulbactam, aztreonam, cefazolin, cefepime, cefotaxime, cefoxitin, ceftazidime, ceftriaxone, ertapenem, imipenem, meropenem, gentamicin, levofloxacin, piperacillin/tazobactam, TMP/SMX
5.	AR0149	*Escherichia*	*coli*	NDM-7, CMY-42	Resistant to: ampicillin, ampicillin/sulbactam, cefazolin, cefepime, cefotaxime, cefoxitin, ceftazidime, ceftriaxone, ertapenem, imipenem, meropenem, levofloxacin, piperacillin/tazobactam
6.	AR0434	*Escherichia*	*coli*	VEB-5; tet(B)	Resistant to: ampicillin, aztreonam, cefazolin, cefepime, cefotaxime, ceftazidime, ceftriaxone, levofloxacin, piperacillin/tazobactam, tetracycline, TMP/SMX
7.	AR0435	*Escherichia*	*coli*	NDM-1, CMY-42, OXA-9, SHV-12, TEM-1A; aac(6')-Ib, aadA5, aph(3')-Ib, aph(6)-Id;	Resistant to: ampicillin, ampicillin/sulbactam, aztreonam, cefazolin, cefepime, cefotaxime, cefoxitin, ceftazidime,

				mph(E), msr(E); sul2; tet(A), tet(R); dfrA12	ceftriaxone, ertapenem, imipenem, meropenem, gentamicin, levofloxacin, piperacillin/tazobactam, tetracycline, TMP/SMX
8.	AR0452	*Escherichia*	*coli*	NDM-5, TEM-1B; tet(B); dfrA12	Resistant to: ampicillin, ampicillin/sulbactam, cefazolin, cefepime, cefotaxime, cefoxitin, ceftazidime, ceftriaxone, ertapenem, imipenem, meropenem, levofloxacin, piperacillin/tazobactam, tetracycline, TMP/SMX
9.	AR0048	*Escherichia*	*coli*	NDM-1, CMY-6, CTX-M-15, OXA-2, TEM-1; aac(6')-Ib3, aadA2, aph(3')-Ia, aph(6)-Id, rmtC, strA; ble-MBL, catA1; ACRF, EMRD, qacEΔ1; sul1; tet(B); dfrA12, dfrA29, dfrA4	Resistant to: ampicillin, ampicillin/sulbactam, aztreonam, cefazolin, cefepime, cefotaxime, cefoxitin, ceftazidime, ceftriaxone, ertapenem, imipenem, meropenem, gentamicin, levofloxacin, minocycline, nitrofurantoin, piperacillin/tazobactam, tetracycline, TMP/SMX
10.	AR0055	*Escherichia*	*coli*	NDM-1, CMY-6, OXA-1; aac(3)-IIa, aac(6')-Ib-D181Y, aadA5, rmtC;	Resistant to: ampicillin, ampicillin/sulbactam, aztreonam, cefazolin, cefepime, cefotaxime,

				mph(A), catB4; ACRF, qacEΔ1; sul1; tet(A), tet(R); dfrA17	cefoxitin, ceftazidime, ceftriaxone, ertapenem, imipenem, meropenem, gentamicin, levofloxacin, piperacillin/tazobactam, tetracycline, TMP/SMX
11.	AR0043	*Klebsiella*	*pneumoniae*	SHV-12; EMRD, KDEA; oqxA, oqxB32	Resistant to ampicillin, ampicillin/sulbactam, aztreonam, cefazolin, cefepime, cefotaxime, cefoxitin, ceftazidime, ceftriaxone, ertapenem, levofloxacin, piperacillin/tazobactam
12.	AR0543	*Escherichia*	*coli*	CTX-M-15, TEM-135; aadA1, ant(2")-Ia; ACRF, EMRD, MDF(A); floR; sul1, sul2; tet(A), tet(R); dfrA36	Resistant to ampicillin, ampicillin/sulbactam, aztreonam, cefazolin, cefepime, cefotaxime, cefoxitin, ceftazidime, ceftriaxone, gentamicin, levofloxacin, piperacillin/tazobactam, tetracycline, TMP/SMX
13.	AR0549	*Escherichia*	*coli*	CTX-M-15, OXA-1; aac(6')-Ib-cr, aadA2, aadA5, aph(3")-Ib, aph(6)-Id; ACRF, EMRD, MDF(A), mph(A), catB4; sul1, sul2; tet(A), tet(B), tet(R); dfrA12, dfrA17	Resistant to ampicillin, ampicillin/sulbactam, aztreonam, cefazolin, cefepime, cefotaxime, cefoxitin, ceftazidime, ceftriaxone, ertapenem, meropenem, levofloxacin, minocycline,

					piperacillin/tazobactam, tetracycline, TMP/SMX
14.	AR0559	*Escherichia*	*coli*	NDM-1, CMY-6, OXA-1; aac(3)-IIa, aac(6')-Ib-cr, aadA5, rmtC; ble-MBL; ACRF, MDF(A), mph(A), catB4; sul1; tet(A), tet(R); dfrA17	Resistant to ampicillin, ampicillin/sulbactam, aztreonam, cefazolin, cefepime, cefotaxime, cefoxitin, ceftazidime, ceftriaxone, ertapenem, meropenem, gentamicin, levofloxacin, plazomicin, piperacillin/tazobactam, tetracycline, TMP/SMX
15.	AR0612	*Escherichia*	*coli*	NDM-7; aph(3')-VI, rmtC; QnrB1; sul1	Resistant to ampicillin, ampicillin/sulbactam, cefazolin, cefepime, cefotaxime, cefoxitin, ceftazidime, ceftriaxone, ertapenem, imipenem, meropenem, piperacillin/tazobactam
16.	AR0616	*Escherichia*	*coli*	NDM-5, CMY-42, TEM-1B; aadA2, rmtB; sul1; dfrA12	Resistant to ampicillin, ampicillin/sulbactam, cefazolin, cefepime, cefotaxime, cefoxitin, ceftazidime, ceftriaxone, ertapenem, imipenem, meropenem, piperacillin/tazobactam, TMP/SMX

17.	AR0147	*Klebsiella*	*oxytoca*	KPC-3, OXY-1-4; aadB, aph(3’)-Ic; sul1; dfrA3b	Resistant to ampicillin, ampicillin/sulbactam, cefazolin, ceftriaxone, TMP/SMX
18.	AR0034	*Klebsiella*	*pneumoniae*	IMP-4, SHV-11, TEM-1; aac(3)-IId, aac(6’)-Ib-G; EMRD, KDEA, qacEΔ1, qacG2; mph(A); catB3; oqxA, oqxB25, QnrB2; sul1	Resistant to ampicillin, ampicillin/sulbactam, cefazolin, cefotaxime, cefoxitin, ceftazidime, ceftriaxone, ertapenem, meropenem, gentamicin, tobramycin, TMP/SMX
19.	AR0854	*Klebsiella*	*pneumoniae*	CTX-M-15, OXA-1, SHV-11, TEM-1; aadA1, aadA5, ant(2”)-Ia, armA; aac(6’)-Ib-cr; EMRD, KDEA, oqxA, oqxB, qacEΔ1; fosA10; mph(E), msr(E); catA1, catB4; QnrS1; sul1; tet(A), tet(R); dfrA1, dfrA17; truncated Omp35, OmpK35	Resistant to ampicillin, ampicillin/sulbactam, aztreonam, cefazolin, cefepime, cefotaxime, cefoxitin, ceftazidime, ceftazidime/avibactam, ceftolozane/tazobactam, ceftriaxone, colistin, gentamicin, levofloxacin, piperacillin/tazobactam, plazomicin, tetracycline, tigecycline, TMP/SMX
20.	AR0860	*Klebsiella*	*pneumoniae*	CTX-M-15, OXA-1, SHV-1, TEM-1; aph(3”)-Ib, aph(6)-Id; aac(6’)-Ib-cr; EMRD, KDEA, oqxA, QnrB1; fosA5; catB4; sul2;	Resistant to ampicillin, ampicillin/sulbactam, aztreonam, cefazolin, cefepime, cefotaxime, cefoxitin, ceftazidime, ceftolozane/tazobactam, ceftriaxone, colistin,

				tet(A), tet(R); dfrA14; truncated Omp36, OmpC, OmpC2, OmpK36	doripenem, ertapenem, meropenem, levofloxacin, piperacillin/tazobactam, tetracycline, TMP/SMX
21.	AR0276	*Acinetobacter*	*baumannii complex*	ADC-239	Resistant to ampicillin/sulbactam, cefepime, cefotaxime, ceftazidime, ceftriaxone, ciprofloxacin, doripenem, gentamicin, levofloxacin, meropenem, piperacillin/tazobactam, tobramycin
22.	AR0312	*Acinetobacter*	*baumannii*	A1, A2, ADC-79, MBL, OXA-69; ant(3")-IIa, aph(3')-Ia; AMVA; catA1; sul2; tet(B)	Resistant to ampicillin/sulbactam, cefepime, cefotaxime, ceftazidime, ceftriaxone, ciprofloxacin, doripenem, gentamicin, levofloxacin, meropenem, piperacillin/tazobactam, tetracycline, tobramycin, TMP/SMX
23.	AR0606	*Klebsiella*	*pneumoniae*	KPC-2, CTX-M-15, OXA-1, SHV-99, TEM-1B; aac(3)-IIa, aac(6')-Ib-cr, aph(3')-Ib, aph(6)-	Resistant to ampicillin, ampicillin/sulbactam, aztreonam, cefazolin, cefepime, cefotaxime, cefoxitin, ceftazidime,

				Id; catB4; QnrB1; sul2; tet(A), tet(R); dfrA14	ceftazidime/avibactam, ceftolozane/tazobactam, ceftriaxone, doripenem, ertapenem, imipenem, meropenem, levofloxacin, piperacillin/tazobactam, tigecycline, tobramycin, tetracycline, TMP/SMX
24.	AR0506	*Klebsiella*	*pneumoniae*	CTX-M-14b, CTX-M-15, NDM-1, OXA-1, OXA-10, SHV-12, TEM-1B; aac(3)-IIa, aac(6')-Il, aph(3')-Ia, aph(3')-Ib, aph(6)-Id; catB4; QnrS1; sul1, sul2; tet(D); dfrA14	Resistant to ampicillin, ampicillin/sulbactam, aztreonam, cefazolin, cefepime, cefotaxime, cefoxitin, ceftazidime, ceftriaxone, ertapenem, imipenem, meropenem, gentamicin, levofloxacin, minocycline, piperacillin/tazobactam, tetracycline, TMP/SMX
25.	AR0553	*Klebsiella*	*pneumoniae*	OXA-181, CTX-M-15, SHV-26; aac(3)-IId, aadA2, armA; EMRD, KDEA; fosA5; sul1, sul2; dfrA12, dfrA14	Resistant to ampicillin, ampicillin/sulbactam, aztreonam, cefazolin, cefepime, cefotaxime, cefoxitin, ceftazidime, ceftriaxone, ertapenem, imipenem, meropenem, gentamicin, levofloxacin, piperacillin/tazobactam, plazomicin, TMP/SMX

26.	AR0555	*Klebsiella*	*pneumoniae*	NDM-5, OXA-232, CTX-M-15, OXA-9, SHV-12, TEM-1A; aac(6')-Ib-G, aadA1, aph(3')-Ia, rmtF1; EMRD, KDEA; mph(A), catB, ARR-2; oqxA, oqxB25, QnrS1; sul1; dfrA14	Resistant to ampicillin, ampicillin/sulbactam, aztreonam, cefazolin, cefepime, cefotaxime, cefoxitin, ceftazidime, ceftriaxone, ertapenem, imipenem, meropenem, gentamicin, levofloxacin, minocycline, nitrofurantoin, omadacycline, piperacillin/tazobactam, tetracycline, TMP/SMX
27.	AR0601	*Klebsiella*	*pneumoniae*	CTX-M-2, OXA-2, SHV-1, TEM-1B; aac(3)-IIa; sul1	Resistant to ampicillin, ampicillin/sulbactam, aztreonam, cefazolin, cefepime, cefotaxime, ceftazidime, ceftriaxone, gentamicin, levofloxacin, piperacillin/tazobactam, TMP/SMX
28.	AR0602	*Klebsiella*	*pneumoniae*	CTX-M-15, OXA-1, SHV-1; aac(6')-Ib-cr, aph(3')-Ia, aph(3')-Ib, aph(6)-Id; fosA; catB4; QnrB1; sul2; dfrA14	Resistant to ampicillin, ampicillin/sulbactam, aztreonam, cefazolin, cefepime, cefotaxime, ceftazidime, ceftriaxone, gentamicin, levofloxacin, piperacillin/tazobactam, tetracycline, TMP/SMX
29.	AR0603	*Klebsiella*	*pneumoniae*	CTX-M-15, SHV-11, TEM-1A; aph(6)-Id; sul2; tet(D); dfrA14	Resistant to ampicillin, ampicillin/sulbactam, aztreonam, cefazolin, cefepime, cefotaxime,

					ceftazidime, ceftriaxone, gentamicin, levofloxacin, piperacillin/tazobactam, tetracycline, TMP/SMX
30.	AR0080	*Klebsiella*	*pneumoniae*	IMP-4, OKP-B-21, OXA-1, SFO-1, TEM-1; aac(3)-IId, aac(6')-Ib-G, aph(3")-Ib, aph(6)-Id; KDEA, qacEΔ1, qacG2; catB3; ARR-3; sul1	Resistant to ampicillin, ampicillin/sulbactam, aztreonam, cefazolin, cefepime, cefotaxime, cefoxitin, ceftazidime, ceftriaxone, ertapenem, imipenem, meropenem, gentamicin, tobramycin, piperacillin/tazobactam
31.	AR0126	*Klebsiella*	*pneumoniae*	KPC-2, OXA-1, TEM-1B; fosA; catB3; oqxA; sul1; dfrA1; truncated OmpK36	Resistant to ampicillin, ampicillin/sulbactam, aztreonam, cefazolin, cefepime, cefotaxime, ceftazidime, ceftriaxone, ertapenem, imipenem, meropenem, gentamicin, levofloxacin, piperacillin/tazobactam, TMP/SMX
32.	AR0135	*Klebsiella*	*pneumoniae*	VIM-1, OXA-9, SHV-12, TEM-1A; aac(3)-IIa, aac(6')-Ib, aph(3')-XV; catB2; oqxA; sul1; tet(D); dfrA14; truncated OmpK35	Resistant to ampicillin, ampicillin/sulbactam, aztreonam, cefazolin, cefepime, cefotaxime, ceftazidime, ceftriaxone, ertapenem, imipenem, meropenem, gentamicin, levofloxacin, piperacillin/tazobactam, tetracycline, TMP/SMX

Supplementary Table 2. List of the organisms included in the ASTar IUO panel

S. No.	Organisms included in the ASTar IUO panel
1.	*Escherichia coli*
2.	*Klebsiella pneumoniae*
3.	*Proteus mirabilis*
4.	*Enterobacter cloacae complex*
5.	*Klebsiella oxytoca*
6.	*Acinetobacter baumannii*
7.	*Serratia marcescens*
8.	*Klebsiella aerogenes*
9.	*Pseudomonas aeruginosa*
