# Supplementary material for: Advancing antimicrobial therapy: evaluating the ASTar (Q-linea) System for rapid AST in Gram-negative bloodstream infections
Source: Microbiol Spectr. 2026 Apr 20;14(6):e03581-25. doi: 10.1128/spectrum.03581-25 (PMC13227951; doi:10.1128/spectrum.03581-25)
Supplement: Table S3 — Isolates with AST discrepancies that underwent resolution testing using Sensititre panels. [file spectrum.03581-25-s0002.pdf]

S. No	Organism
**1**	*Proteus mirabilis*
**2**	*Proteus mirabilis*
**3**	*Serratia marcescens*
**4**	*Enterobacter cloacae complex*
**5**	*Proteus mirabilis*
**6**	*Escherichia coli*
**7**	*Proteus mirabilis*

Supplementary Table 3: Isolates with AST discrepancies that underwent resolution testing using Sensititre panels.
