# Supplementary material for: Advancing antimicrobial therapy: evaluating the ASTar (Q-linea) System for rapid AST in Gram-negative bloodstream infections
Source: Microbiol Spectr. 2026 Apr 20;14(6):e03581-25. doi: 10.1128/spectrum.03581-25 (PMC13227951; doi:10.1128/spectrum.03581-25)
Supplement: Tables S4 and S5 — Table S4: Essential agreement, categorical agreement, very major error, and major error rates by antimicrobial agent for ASTar vs Standard-of-Care method for 76 prospective clinical specimens, after discrepancy resolution. Table S5: Essential agreement, categorical agreement, very major error, and major error rates by antimicrobial agent for ASTar vs Standard-of-Care method for 32 CDC & FDA AR Isolate Bank samples, after discrepancy resolution. [file spectrum.03581-25-s0003.pdf]

	EA (n/N)			CA (n/N)			VME		ME	
**Antibiotics**	**EA % (n/N)**	**EA (n)**	**EA (N)**	**CA % (n/N)**	**CA (n)**	**CA (N)**	**VME %**	**VME (n)**	**ME %**	**ME (n)**
Amikacin	100.0%	24	24	100.0%	24	24	0.0%	0	0.0%	0
Ampicillin	100.0%	50	50	100.0%	50	50	0.0%	0	0.0%	0
Ampicillin-sulbactam	98.5%	67	68	98.5%	67	68	0.0%	0	0.0%	0
Aztreonam	95.8%	23	24	100.0%	24	24	0.0%	0	0.0%	0
Cefazolin	97.1%	67	69	98.6%	68	69	0.0%	0	0.0%	0
Cefepime	93.4%	71	76	96.1%	73	76	0.0%	0	0.0%	0
Cefotaxime	100.0%	23	23	100.0%	23	23	0.0%	0	0.0%	0
Ceftazidime	100.0%	74	74	100.0%	74	74	0.0%	0	0.0%	0
Ceftazidime-avibactam	100.0%	23	23	100.0%	24	24	0.0%	0	0.0%	0
Ceftriaxone	98.6%	73	74	98.6%	73	74	0.0%	0	1.7%	1
Cefuroxime	100.0%	70	70	100.0%	70	70	0.0%	0	0.0%	0
Ciprofloxacin	96.0%	24	25	96.0%	24	25	0.0%	0	0.0%	0
Ertapenem	100.0%	2	2	100.0%	2	2	0.0%	0	0.0%	0
Gentamicin	97.4%	74	76	100.0%	76	76	0.0%	0	0.0%	0
Levofloxacin	100.0%	75	75	100.0%	75	75	0.0%	0	0.0%	0
Meropenem	100.0%	74	74	100.0%	74	74	0.0%	0	0.0%	0
Meropenem-vaborbactam	100.0%	23	23	100.0%	23	23	0.0%	0	0.0%	0
Piperacillin-tazobactam	100.0%	75	75	100.0%	75	75	0.0%	0	0.0%	0
Tigecycline	100.0%	20	20	100.0%	20	20	0.0%	0	0.0%	0
Tobramycin	98.7%	75	76	98.7%	75	76	0.0%	0	0.0%	0
Trimethoprim-sulfamethoxazole	96.0%	72	75	100.0%	75	75	0.0%	0	0.0%	0
**Grand Total**	**98.4%**	**1079**	**1096**	**99.3%**	**1089**	**1097**	**0.0%**	**0**	**0.1%**	**1**

Supplementary Table 4: Essential Agreement (EA), Categorical Agreement (CA), Very Major Error (VME), and Major Error (ME) rates by antimicrobial agent for ASTar Vs Standard-of-Care method for 76 prospective clinical specimens, after discrepancy resolution; n= number of agreements and N= number of tested isolates

	EA (n/N)			CA (n/N)			VME		ME	
**Antibiotics**	**EA % (n/N)**	**EA (n)**	**EA (N)**	**CA % (n/N)**	**CA (n)**	**CA (N)**	**VME %**	**VME (n)**	**ME %**	**ME (n)**
Amikacin	96.9%	31	32	96.9%	31	32	10.0%	1	0.0%	0
Ampicillin	100.0%	14	14	100.0%	14	14	0.0%	0	0.0%	0
Ampicillin-sulbactam	100.0%	32	32	100.0%	32	32	0.0%	0	0.0%	0
Aztreonam	96.4%	27	28	96.4%	27	28	0.0%	0	0.0%	0
Cefazolin	100.0%	29	29	100.0%	29	29	0.0%	0	0.0%	0
Cefepime	100.0%	29	29	100.0%	29	29	0.0%	0	0.0%	0
Cefotaxime	93.8%	30	32	100.0%	32	32	0.0%	0	100.0%	1
Cefoxitin	100.0%	16	16	100.0%	16	16	0.0%	0	0.0%	0
Ceftazidime	96.9%	31	32	96.9%	31	32	0.0%	0	100.0%	1
Ceftazidime-avibactam	96.0%	24	25	96.0%	24	25	0.0%	0	9.1%	1
Ceftolozane-tazobactam	92.9%	13	14	92.9%	13	14	0.0%	0	0.0%	0
Ceftriaxone	93.1%	27	29	100.0%	29	29	0.0%	0	0.0%	0
Ciprofloxacin	100.0%	30	30	100.0%	30	30	0.0%	0	0.0%	0
Ertapenem	86.7%	13	15	86.7%	13	15	16.7%	2	0.0%	0
Gentamicin	96.7%	29	30	96.7%	29	30	0.0%	0	11.1%	1
Levofloxacin	100.0%	30	30	100.0%	30	30	0.0%	0	0.0%	0
Meropenem	71.9%	23	32	84.4%	27	32	5.9%	1	0.0%	0
Meropenem-vaborbactam	100.0%	1	1	100.0%	1	1	0.0%	0	0.0%	0
Piperacillin-tazobactam	96.7%	29	30	100.0%	30	30	0.0%	0	0.0%	0
Tigecycline	100.0%	29	29	100.0%	29	29	0.0%	0	0.0%	0
Tobramycin	90.0%	27	30	90.0%	27	30	0.0%	0	0.0%	0
Trimethoprim-sulfamethoxazole	96.7%	29	30	96.7%	29	30	8.0%	2	0.0%	0
**Grand Total**	**95.4%**	**543**	**569**	**97.0%**	**552**	**569**	**1.4%**	**6**	**3.7%**	**4**

Supplementary Table 5: Essential Agreement (EA), Categorical Agreement (CA), Very Major Error (VME), and Major Error (ME) rates by antimicrobial agent for ASTar Vs Standard-of-Care method for 32 CDC & FDA AR Isolate Bank samples, after discrepancy resolution; n= number of agreements and N= number of tested isolates.
